# Preparation and Evaluation of Dexamethasone-Loaded Electrospun Nanofiber Sheets as a Sustained Drug Delivery System

**DOI:** 10.3390/ma9030175

**Published:** 2016-03-08

**Authors:** Jin Woo Lee, Hye Yun Lee, Seung Hun Park, Ji Hoon Park, Jae Ho Kim, Byoung Hyun Min, Moon Suk Kim

**Affiliations:** 1 Department of Applied Chemistry and Biological Engineering, Ajou University, Suwon 443-759, Korea; dlwlswlsdndn@ajou.ac.kr (J.W.L.); leeyn3679@ajou.ac.kr (H.Y.L.); jhkim@ajou.ac.kr (J.H.K.); 2 Department of Molecular Science and Technology, Ajou University, Suwon 443-759, Korea; hpt88@ajou.ac.kr (S.H.P.); jhp@ajou.ac.kr (J.H.P.); bhmin@ajou.ac.kr (B.H.M.)

**Keywords:** electrospinning, nanofiber sheets, dexamethasone, sustained delivery, biodegradation

## Abstract

Recently, electrospinning technology has been widely used as a processing method to make nanofiber sheets (NS) for biomedical applications because of its unique features, such as ease of fabrication and high surface area. To develop a sustained dexamethasone (Dex) delivery system, in this work, poly(*ε*-caprolactone-co-l-lactide) (PCLA) copolymer with controllable biodegradability was synthesized and further utilized to prepare electrospun Dex-loaded NS using water-insoluble Dex (Dex(b)) or water-soluble Dex (Dex(s)). The Dex-NS obtained by electrospinning exhibited randomly oriented and interconnected fibrillar structures. The *in vitro* and *in vivo* degradation of Dex-NS was confirmed over a period of a few weeks by gel permeation chromatography (GPC) and nuclear magnetic resonance (NMR). The evaluation of *in vitro* and *in vivo* Dex(b) and Dex(s) release from Dex-NS showed an initial burst of Dex(b) at day 1 and, thereafter, almost the same amount of release as Dex(b) for up to 28 days. In contrast, Dex(s)-NS exhibited a small initial burst of Dex(s) and a first-order releasing profile from Dex-NS. In conclusion, Dex-NS exhibited sustained *in vitro* and *in vivo* Dex(s) release for a prolonged period, as well as controlled biodegradation of the NS over a defined treatment period.

## 1. Introduction

Nanofiber structures exhibit the distinctive physical and mechanical properties of nano-scaled materials [1]. Nanofibers can be produced using several techniques, such as phase separation, self-assembly, electrospinning, *etc.* [2].

Among several techniques, electrospinning is one of the most simple and cost-effective methods for the preparation of nanofiber sheets (NS) [3]. During the last several decades, there has been a remarkable growth in the development of electrospun NS for biomedical applications. NS can be used in various biomedical applications such as drug delivery, wound healing, reinforced biomaterials, scaffolds for tissue engineering, *etc.* [3,4,5,6].

The preparation of electrospun NS for biomedical applications requires the selection of a suitable polymer. Various types of natural and synthetic materials have been used to fabricate electrospun NS. These include synthetic materials such as poly(lactic acid) (PLA), poly(glycolide) (PGA), poly(d,l-lactic-co-glycolic acid) (PLGA), poly(*ε*-caprolactone) (PCL), *etc.* and natural polymers such as chitosan, gelatin, collagen, *etc.* [3,4,5,6,7,8,9,10].

PCL is one of the most widely used synthetic materials in electrospinning due to its favorable mechanical and biodegradable properties [11]. However, PCL is limited by its degradation kinetics being slower than that of PLA, PGA, or PLGA [12].

In previously published work, we reported the controllable biodegradability and excellent biocompatibility of a poly(*ε*-caprolactone-co-l-lactide) (PCLA) random copolymer [13,14,15]. The PCLA can ideally degrade over a period ranging from days to a few weeks [13]. In addition, PCLA has already been approved by the US Food and Drug Administration and Conformit Europe for clinical biomedical application [16]. Thus, the first aim of this work was the fabrication of an NS with several potential advantages such as ease preparation and *in vivo* controllable biodegradability by electrospinning of PCLA.

In general, the drug release from a drug carrier could depend on the penetrability of biologic fluid inside the drug carrier and/or biodegradation of the drug carrier. Considering the penetrability of biologic fluid, the drug structure can change penetrability of biologic fluid and, consequently, the wettability of the drug carrier, which is known to be of importance for the controlled release of drug [17].

Dexamethasone (Dex(b)) is a type of synthetic glucocorticoid steroid [18], while dexamethasone 21-phosphate disodium salt (Dex(s)) is a phosphate ester prodrug of Dex [19]. Dex(s) is water-soluble due to the two ionizable phosphate groups. Both Dex(b) and Dex(s) are widely used as an anti-inflammatory and immunosuppressant drug. In addition, Dex is used as an adjunctive therapy for short-term administration in endocrine and rheumatic disorders. Recently, the activity of Dex in the treatment of cancer was demonstrated [20]. Thus, the second aim of this work was the comparison of the release behavior of Dex(b) and Dex(s).

**Figure 1:**
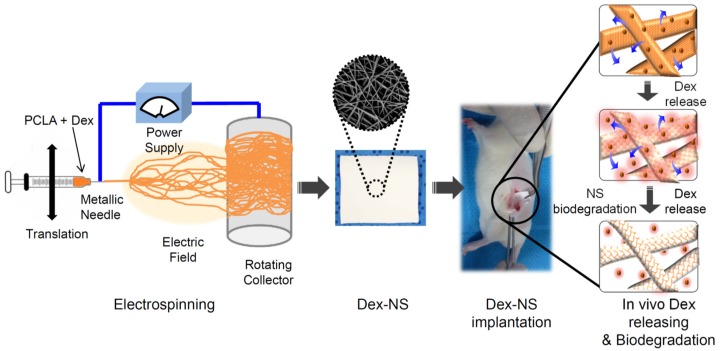
Schematic showing the experimental procedures of preparation, implantation, *in vivo* release and *in vivo* biodegradation of Dex-NS.

Owing to its wide therapeutic application potential, it is interesting to develop a Dex delivery system suited for a long-term administration of Dex. Thus, a sustained Dex delivery system can offer a number of potentially important clinical advantages, including significantly reduced dosage frequency and improved efficacy [21,22,23,24]. In light of this, the third aim of this work was to prepare Dex-loaded NS (Dex-NS) as a sustained Dex(b) and Dex(s) delivery system. The application of Dex-NS represents a promising approach to maintain concentrations of Dex within implanted positions for prolonged periods, as well as to achieve biodegradation of the NS depot over a defined period (Figure 1). Answers to these questions will enable feasible development of suitable NS as a sustained Dex delivery system.

## 2. Materials and Methods

### 2.1. Materials

The methoxy poly(ethylene glycol*)* (MPEG) (number-average molecular weight (*M_n_* = 750), and Sn(Oct)_2_ (Aldrich, Yongin, Korea) were used as received. *ε*-Caprolactone (CL) was distilled over CaH_2_ under reduced pressure. l-Lactide (LA; Boehringer Ingelheim, Blanquefort, France) was recrystallized twice in ethyl acetate. Dexamethasone (Dex(b)) and dexamethasone 21-phosphate disodium salt (Dex(s)) were purchased from TCI (Tokyo, Japan) and Sigma-Aldrich (St. Louis, MO, USA), respectively.

### 2.2. Characterization

Proton nuclear magnetic resonance (^1^H-NMR) spectra were measured using a Varian Mercury Plus 400 MHz instrument (Varian, CA, USA) with deuterated chloroform (CDCl_3_) in the presence of tetramethylsilane (TMS) as an internal standard. The original PCLA and PCLA separated from Dex-NS after *in vitro* and *in vivo* degradation were solubilized in anhydrous chloroform (CHCl_3_). Molecular weight was measured using an YL-Clarity GPC (gel permeation chromatograph) system (YL 9170 RI detector, YL Instruments, Anyang, Korea) with three columns (Shodex K-802, K-803, and K-804 polystyrene gel columns) at 40 °C by polystyrene calibration and using anhydrous CHCl_3_ as an eluent with a flow rate of 1.0 mL/min.

### 2.3. Synthesis of PCLA Random Copolymer

All glasses were heated in a vacuum and flushed with a dry nitrogen stream for drying. The polymerization process to produce PCLA with a CL/LA ratio of 40/60 and molecular weight of 350,000 g/mol using MPEG (750 g/mol) as an initiator is as follows. MPEG (0.03 g, 0.04 mM) and toluene (80 mL) were added into a flask. Azeotropic distillation was performed to remove water from the MPEG and toluene. Under a dry nitrogen stream, toluene was distilled off to obtain a final volume of 50 mL. CL (4.84 g, 42.4 mM) and LA (9.16 g, 63.6 mM) were introduced to the MPEG solution at room temperature, followed by the addition of 0.5 mL of Sn(Oct)_2_ solution (0.1 M in dried toluene). The mixture was reacted at 130 °C for 36 h and then poured into a mixture of *n*-hexane and ethyl ether (*v/v* = 4/1) to precipitate a polymer. The precipitated polymers were obtained from the supernatant by decantation, dissolved in CH_2_Cl_2_, and then filtered. The resulting polymer solution was concentrated by rotary evaporation and dried in a vacuum to yield a colorless polymer. The molecular weight of PCLA copolymer was determined by comparing the intensity of the signal in ^1^H-NMR spectroscopy.

### 2.4. Preparation of Dex-NS

The solution of PCLA (825 mg) and Dex(b) or Dex(s) (2.75, 5.5 and 11 mg) were prepared in 5.5 mL of chloroform and methanol (10:1 *v/v*). The viscosities of solution formulation were 1.4 ± 0.6~1.6 ± 0.7 Pa·s in a rheometer. By using the prepared solution formulation, Dex-loaded NS (Dex-NS) was prepared as the Dex(b) or Dex(s), with concentrations of 0.5, 1, and 2 mg. The electrospinning of Dex-NS was performed at a voltage of 15 kV using a homemade ES-1 (DaeLim Starlet, Siheung, Korea). The distance between the needle tip and the collector was 10 cm. The flow rate of the PCLA and Dex solution was maintained at 3 mL/h from the 24-G needle outlet by using a syringe pump. Grounded aluminum foil was used as a collector as shown in Figure 1. The Dex-NS was collected from the surface of the aluminum foil, dried in vacuum, and stored in a vacuum desiccator. For scanning electron microscopy (SEM) measurements, the Dex-NS was coated with a conductive layer of gold using a plasma-sputtering apparatus (Emitech, K575, Kent, UK). The SEM image was obtained using an FE-SEM (JSM-6700F, JEOL, Tokyo, Japan). Dex-NS was cut into discs with 12 mm diameters and with weights of 3.7 ± 0.55 mg. Dex-NS was transferred onto silicone wafer which was washed with MeOH. AFM measurements were carried out in non-contact mode with Atomic Force Microscopy (AFM, Advanced Scanning Probe Microscope, XE-100, PSIA, Park system, Suwon, Korea) with image processing software (Park System, Suwon, Korea). The encapsulation efficiency was determined using the following equation: E (%) = [(amount of Dex obtained in Dex-NS)/(initial amount of Dex added in Dex-NS)] × 100.

### 2.5. In Vitro Degradation Test

The Dex-NS were immersed in 4 mL of phosphate-buffered saline (PBS) in a vial (5 mL) and incubated at 37 °C with shaking at 100 rpm for a period of 1 week, 2 weeks, and 3 weeks (*n* = 3 for each data point). At determined time intervals, the vials were retrieved and freeze-dried for 3 days. Immediately after freeze-drying, SEM images were observed, and the *M*_w_ change was measured using a GPC system.

### 2.6. In Vitro Release Study

The Dex(b)-NS and Dex(s)-NS were immersed in 4 mL PBS in a vial (5 mL) and incubated at 37 °C with shaking at 100 rpm for a period of 28 days. For each experiment, 0.5 mL of the sample was extracted from the vial at determined time intervals; 0.5 mL pure PBS was immediately added into the vial. The amount of Dex(b) and Dex(s) was analyzed using a high-performance liquid chromatography (HPLC) system (Agilent 1200 series, Waldbronn, Germany) equipped with detection at 220 nm. The RP18 column (150 mm × 3.9 mm inner diameter (i.d.), 5 µm particle size) for Dex(b) and hypersil C18 column (250 mm × 4.6 mm i.d., 5 µm particle size) for Dex(s) were used. The mobile phase was prepared as a mixture of PBS and acetonitrile (ACN) at a ratio of 50:50 (*v/v*) for Dex(b) and 60:40 (*v/v*) for Dex(s). The mobile phase was eluted at a flow rate of 1.0 mL/min. Four independent release experiments were performed for each Dex-NS. Eluents were identified by comparing their peaks to the retention time of pure standards and quantified by UV absorption peak area at 254 nm.

### 2.7. In Vivo Implantation

The protocols of this study were approved by the Institutional Animal Experiment Committee at the Ajou University School of Medicine (No. 2014-0051). Twelve 6-week-old Sprague-Dawley (SD) rats (270–350 g) were randomly assigned into two groups. Each rat was anesthetized with zoletil and rompun (1:1 ratio, 2 mL/kg). The Dex-NS (*n* = 4 for each rat; *n* = 3 for releasing test, *n* = 1 for NMR and GPC measurements) were implanted subcutaneously under the dorsal skin and allowed to develop *in vivo* over 3 weeks. At each of the post-implantation sampling points, the rats were sacrificed, and the Dex-NS were removed individually from the subcutaneous dorsum.

To determine drug release, Dex(b)-NS and Dex(s)-NS were individually placed in a test tube. The Dex(b)-NS was dissolved by the addition of CH_2_Cl_2_ (2 mL). The CH_2_Cl_2_ solution was isolated and removed through an evaporator. Then, the mixture was added into MeOH to separate PCLA and Dex(b). The Dex(s)-NS was also dissolved by the addition of CH_2_Cl_2_ (2 mL). Then, 2 mL of deionized water (DW) was introduced to solubilize the Dex(s). The contents of Dex(b) and Dex(s) were determined using HPLC described in Section 2.6. The amount of Dex released *in vivo* was determined as (initial amount of Dex in Dex-NS)−(the Dex remaining in the Dex-NS removed from rats at the determined time).

For NMR measurement, the removed Dex-NS was solubilized in 1 mL of CDCl_3_ and sonicated for 60 min at 25 °C. The CDCl_3_ solution was collected through filtration and was determined by ^1^H-NMR spectroscopy.

For GPC measurement, the removed Dex-NS was solubilized in 1 mL of CHCl_3_ and sonicated for 60 min at 25 °C. The CHCl_3_ solution was collected via filtration, dried in an oven for 60 min and evaluated using GPC.

## 3. Results and Discussion

### 3.1. Preparation of PCLA Copolymers

**Scheme 1:**
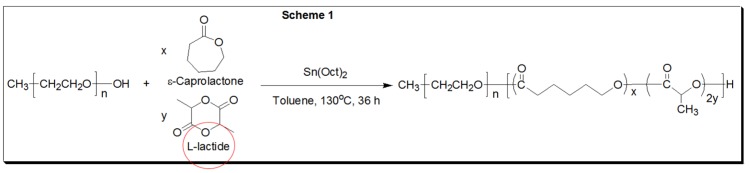
Synthesis scheme of PCLA copolymers.

The current study was to apply PCLA as the controllable biodegradable material to develop NS using electrospinning. Our previous work demonstrated the controllable biodegradability of PCLA over a period ranging from days to a few weeks [13,14]. The degradation can be controlled by varying attributes such as the molecular weight and composition of polyesters. Based on previous results, we designed the PCLA copolymer with a molecular weight of 350,000 g/mol and composition having CL/LA ratio of 40/60 (Scheme 1).

**Figure 2:**
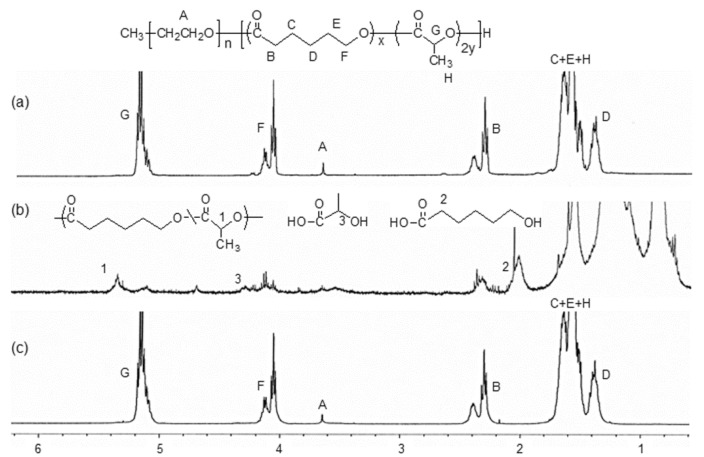
Proton nuclear magnetic resonance (^1^H-NMR) spectra (**a**) before; and (**b** and **c**) after 3 weeks of *in vitro* degradation of Dex-NS; (**b**) *n*-hexane and ethyl ether soluble portions; and (**c**) insoluble portions.

The PCLA copolymer was prepared by ring opening of CL and LA monomer using MPEG as an initiator in the presence of Sn(Oct)_2_. The molecular weight of the PCLA copolymer and ratio of the CL and LA units in the PCLA copolymer were determined using ^1^H-NMR spectra by comparing the intensity of the total proton signal *A* of MPEG as an initiator and the methylene proton signals (*G* and *F*) for 5.15 and 3.95–4.2 ppm of PLLA and PCL, respectively (Figure 2a). The molecular weight and composition of PLLA and PCL was in agreement with that of the designed composition. Thus, the PCLA copolymer was prepared successfully as a material for the electrospinning experiments.

### 3.2. Preparation of Electrospun Dex-NS

To prepare the Dex-NS, PCLA and Dex(b) or Dex(s) were solubilized in chloroform and methanol. The electrospinning of Dex-NS was performed as shown in Figure 1. The obtained Dex-NS exhibited randomly oriented and interconnected fibrillar structures from SEM and AFM as shown in Section 3.4. The loading efficiencies and diameters of electrospun NS, Dex(b)-NS, and Dex(s)-NS are summarized in Table 1.

### 3.3. In Vitro Degradation of Electrospun Dex-NS

**Figure 3:**
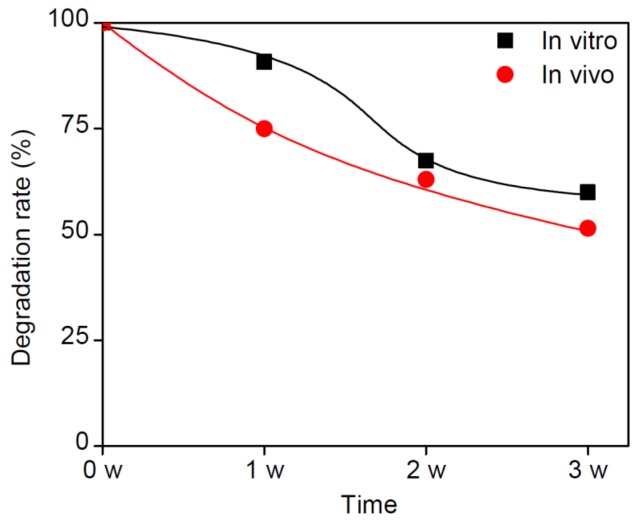
Plot of degraded PCLA as a function of time; the molecular weight of the remained PCLA inside Dex-NS at *in vitro* and *in vivo* were determined by ^1^H-NMR.

The *in vitro* degradation behavior of NS, Dex(b)-NS and Dex(s)-NS was examined in PBS at 37 °C for 3 weeks. Their degradation was examined using NMR, GPC, and SEM. Figure 2 shows the ^1^H-NMR change before and after *in vitro* degradation of Dex-NS. In the soluble portion (Figure 2b), the peaks *1*, *2*, and *3* assignable to degraded oligomers, 6-hydroxylhexanoic, and lactic acid appeared at 5.2 ppm, 4.2 ppm, and 1.95 ppm, respectively. This result indicated that the degraded products were the oligomeric (*ε*-caprolactone-co-l-lactide) as well as the LA and CL derivatives used in the polymerization of PCLA. The molecular weight of the remained PCLA inside Dex-NS was determined by NMR. The *in vitro* degraded amounts of PCLA *versus* time were plotted and are shown in Figure 3. The *in vitro* degradation of Dex-NS gradually occurred as a function of time. The *in vivo* degradation was slightly faster than *in vitro* degradation.

**Figure 4:**
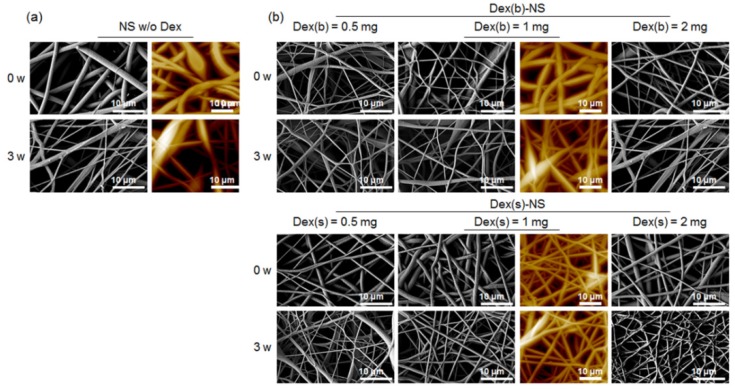
SEM images before and after *in vitro* biodegradation of (**a**) NS; (**b**) Dex(b)-NS, and Dex(s)-NS with Dex 0.5–2 mg (SEM: 3000 × magnification, AFM: 40 μm × 40 μm).

Figure 4 showed the SEM images before and after *in vitro* degradation. There was a small difference in the morphology before and after degradation of NS, Dex(b)-NS, and Dex(s)-NS. The images showed roughly swelled fibrillar structures of NS, Dex(b)-NS, and Dex(s)-NS. The diameter before and after *in vivo* degradation of NS, Dex(b)-NS and Dex(s)-NS were measured by AFM. The diameters changed from about 500–700 nm to 700–990 nm at 3 weeks, indicating the swelled NS.

**Figure 5:**
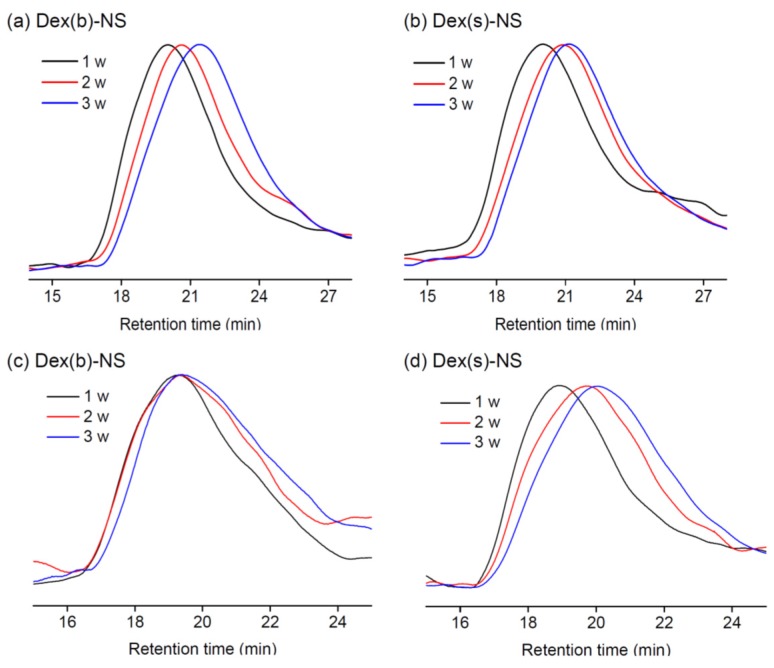
Gel permeation chromatograph (GPC) charts to analyze (**a**,**b**) *in vitro*; and (**c**,**d**) *in vivo* degradation of (**a**,**c**) Dex(b)-NS; and (**b**,**d**) Dex(s)-NS for 1–3 weeks.

The degradation of Dex-NS was also monitored using GPC (Figure 5a,b). The molecular weight peak of Dex-NS shifted to a low value implying the degradation. The *in vitro* degradation of Dex(b)-NS showed a gradual shift in GPC maximum peak from 1 to 3 weeks, while Dex(s)-NS showed a slightly fast shift from 1 to 2 weeks and a slow shift at 3 weeks. Generally, the ester bond linkages of PCLA are degraded by the hydrolytic attack of water molecules. Thus, the degradation rate could be contributed by the water soluble Dex, because Dex(s) had higher water absorption than Dex(b). It was likely that Dex(s)-NS can absorb more water inside NS compared to Dex(b)-NS, likely inducing fast, slight degradation of Dex(s)-NS [25].

### 3.4. In Vitro Dex release from Dex-NS

**Figure 6:**
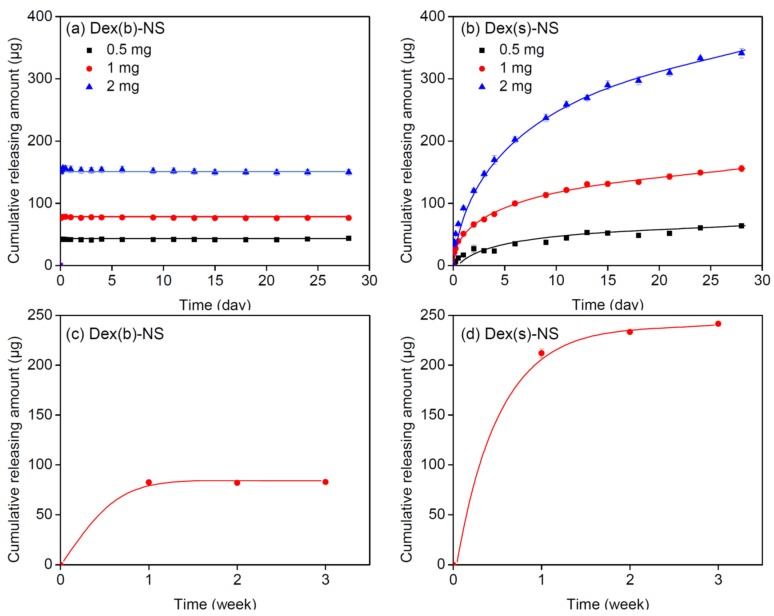
The amount of Dex released from (**a**,**c**) Dex(b)-NS; and (**b**,**d**) Dex(s)-NS. (**a**,**b**) *in vitro*; and (**c**,**d**) *in vivo*.

To evaluate the *in vitro* Dex(b) and Dex(s) release, Dex(b)-NS and Dex(s)-NS were incubated in PBS at 37 °C for 28 days. Figure 6a,b show the Dex release plots for the cumulative released amounts *versus* time.

As shown in Figure 6a, the cumulative *in vitro* Dex(b) releasing from Dex(b)-NS was approximately 40, 80, and 150 µg at 1 day respectively, implying the initial burst of Dex(b) from the surface of the Dex(b)-NS. Then, the release was maintained at almost the same amount as Dex(b) for up to 28 days, indicating little or no release of Dex(b).

Meanwhile, for each of the different Dex(s) concentrations, the cumulative amount of Dex(s) released from the Dex(s)-NS was approximately 63, 155, and 340 µg at 28 days (Figure 6). The Dex(s) release from Dex(s)-NS maintained a sustained release over extended experimental periods. Importantly, there was a limited initial burst of Dex(s) at all tested Dex(s) concentrations. In addition, the release pattern was that of a first-order releasing profile for all concentrations. This demonstrated the sustained release of Dex(s) from the Dex(s)-NS over extended experimental periods.

### 3.5. In Vivo Degradation of Electrospun Dex-NS

To assess the *in vivo* degradation behavior of Dex-NS, Dex(b)-NS and Dex(s)-NS were implanted into SD rats and allowed to develop for 3 weeks. The Dex(b)-NS and Dex(s)-NS were excised at 1, 2, and 3 weeks after implantation and were examined by NMR, GPC, and SEM. In addition, the remained Dex inside Dex-NS was examined to determine the *in vivo* released amount of Dex.

**Figure 7:**
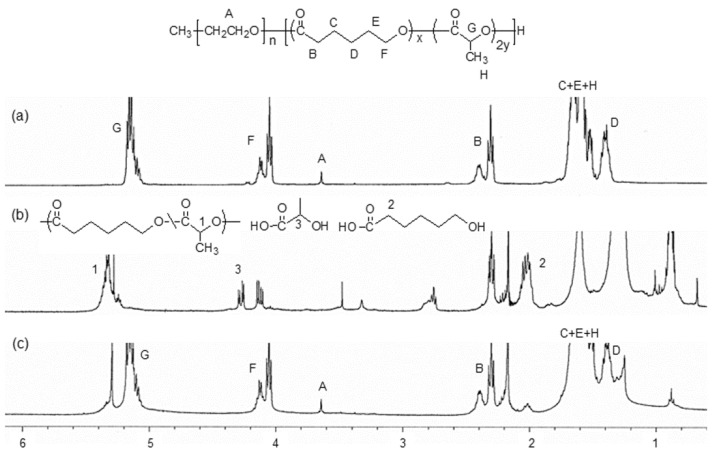
Proton nuclear magnetic resonance (^1^H-NMR) spectra (**a**) before; and (**b** and **c**) after removal of *in vivo* implants from rats after 3 weeks; (**b**) *n*-hexane and ethyl ether soluble portions; and (**c**) insoluble portions.

In the ^1^H-NMR before and after *in vivo* degradation of Dex-NS (Figure 7b), the peaks 1, 2, and 3 were observed at 5.2 ppm, 4.2 ppm, and 1.95 ppm, assignable to degraded oligomers, 6-hydroxylhexanoic, and lactic acid, respectively. The *in vivo* degraded products were in agreement with those of *in vitro* degradation. The *in vivo* degradation of Dex-NS gradually occurred as a function of time (Figure 3). *In vivo* degradation rate of Dex-NS was faster than the *in vitro* rate, likely due to several biologic conditions.

**Figure 8:**
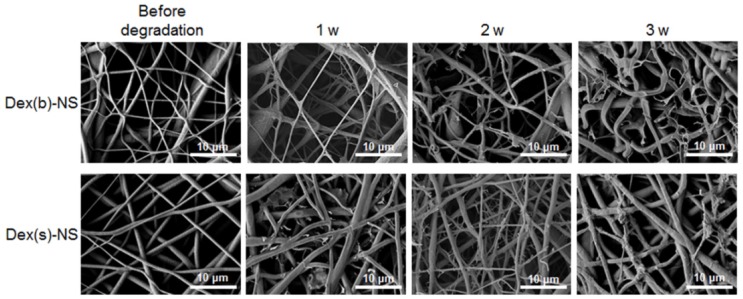
SEM images before and after *in vivo* biodegradation of NS, Dex(b)-NS, and Dex(s)-NS after 1–3 weeks (×3000 magnification).

Figure 8 showed the SEM images before and after *in vivo* degradation of Dex(b)-NS and Dex(s)-NS. Both Dex(b)-NS and Dex(s)-NS exhibited the swelled and degraded fibrillar structures.

In the GPC (Figure 5c,d), the Dex(b)-NS showed peaks at high retention time corresponding to the degradation species in the Dex(b)-NS, but the main peak of the Dex(b)-NS was maintained at the same time. Meanwhile, Dex(s)-NS showed a gradual shift in GPC maximum peak from 1 week to 3 weeks. Dex(s)-NS showed large amounts of low-molecular-weight peaks, even after 2 weeks, indicative of an extensive degradation. This result indicated the difference in *in vivo* degradation between Dex(b)-NS and Dex(s)-NS due to the water solubility of the loaded Dex. Dex(s) inside Dex-NS easily absorb the biologic fluid compared with Dex(b), likely leading to faster hydrolysis of Dex(s)-NS because of better accessibility of biologic fluid to the ester bonds of NS.

### 3.6. In Vivo Dex Release from Dex-NS

The final aim of this work was the development of a degradable Dex-NS for sustained Dex delivery. Our work demonstrated the preparation of biodegradable NS, ideally tailored to match the required Dex release rate and degradation.

Dex(b)-NS and Dex(s)-NS were implanted into SD rats and allowed to develop for 3 weeks. Figure 6c,d show the plots for the *in vivo* cumulative release amounts at 1, 2 and 3 weeks. The amount of Dex(b) released from Dex(b)-NS after 1 week was 70 µg, and the same amount was maintained even at 2 and 3 weeks, indicating little or no *in vivo* release of Dex(b). This was similar to the *in vitro* releasing pattern of Dex(b) from Dex(b)-NS.

The initial burst release of Dex(b)-NS is likely because the Dex(b) in the surface of Dex(b)-NS is rapidly perfused from the NS under *in vivo* condition, although this does not provide an exact explanation for the initial burst. However, because most of the Dex(b) present inside the slightly swelled NS is not easily available to the biological media, the release of Dex(b) maintained a very small amount for up to 28 days.

On the contrary, the cumulative amount of Dex(s) released was 210, 230, and 240 µg from the Dex(s)-NS at 1, 2 and 3 weeks, respectively (Figure 6d). Importantly, the release pattern was that of a first-order releasing profile even at *in vivo* condition. Released concentration of Dex(s) at each time point was about 200 µg, 20 µg and 10 µg at 1 week, 2 weeks and 3 weeks, assignable to about 11 µg/mL, 1 µg/mL and 0.5 µg/mL per blood of SD rat, respectively. Although the concentration did not directly coincide, the released Dex concentration can be effectively applied in specific medical applications [26].

This demonstrated the sustained release of Dex(s) from the Dex(s)-NS for 3 weeks and the similar releasing pattern of Dex(s) from Dex(s)-NS between *in vitro* and *in vivo*. Taken together, we successfully confirmed the possibility of using Dex(s)-NS as a sustained Dex delivery system in agreement with other results [21,22,23], even though we could not provide the exact explanation of the initial burst difference between *in vitro* and *in vivo*.

## 4. Conclusions

In the present study, we successfully manufactured Dex-NS using PCLA with controllable biodegradability. Dex-NS exhibited sustained Dex(s) release within implanted positions for prolonged periods, as well as showed controlled biodegradation over a defined implanted period. Thus, we successfully developed a feasible Dex-NS for a sustained Dex delivery system. Further experiments are necessary to understand the detailed mechanisms for a first-order releasing profile and to investigate the *in vivo* feasibility for specific diseases in animal models.

## Figures and Tables

**Table 1:** Loading efficiencies and diameters of electrospun NS, Dex(b)-NS, and Dex(s)-NS.

Dex-NS	Added Dex Amount (mg)	Obtained Dex Amount (mg)	Loading Efficiency (%)	Dex/PCLA w/w%	Diameter (nm) ^a^
NS	-	-	-	-	621 ± 35
Dex(b)-NS 0.5 mg	2.75	1.73	62.9	0.2	492 ± 81
Dex(b)-NS 1 mg	5.5	3.63	66.0	0.4	513 ± 70
Dex(b)-NS 2 mg	11	8.33	75.7	1.0	524 ± 77
Dex(s)-NS 0.5 mg	2.75	1.82	66.2	0.2	611 ± 43
Dex(s)-NS 1 mg	5.5	3.47	63.1	0.4	688 ± 33
Dex(s)-NS 2 mg	11	8.17	74.3	1.0	601 ± 45

^a^ The diameters of NS, Dex(b)-NS, and Dex(s)-NS were determined by SEM images, following of the analysis using ImageJ (Java version) National Institutes of Health (NIH) image software (NIH, MD, USA).

## Abbreviations

NS	Nanofiber Sheets
Dex	Dexamethasone
Dex(b)	Water-insoluble Dexamethasone
Dex(s)	Water-soluble Dexamethasone
PCLA	Poly(*ε*-caprolactone-co-l-lactide)
PCL	Poly(*ε*-caprolactone)
PLA	Poly(lactic acid)
PLLA	Poly(l-lactide)
PGA	Poly(glycolide)
PLGA	Poly(d,l-lactic-co-glycolic acid)
MPEG	Methoxy Poly(ethylene glycol)
M*_n_*	Number-average Molecular Weight
M*_w_*	Molecular Weight
CL	*ε*-Caprolactone
LA	l-lactide
NMR	Nuclear Magnetic Resonance
GPC	Gel Permeation Chromatograph
SEM	Scanning Electron Microscopy
FE-SEM	Field Emission Scanning Electron Microscopy
PBS	Phosphate Buffered Saline
ACN	Acetonitrile
HPLC	High Performance Liquid Chromatography
SD	Sprague Dawley
